# Exploring potential induction of grapevine (*Vitis* spp.) root phenolic compounds by ring nematodes, *Mesocriconema xenoplax*

**DOI:** 10.1186/s13104-022-06262-2

**Published:** 2022-12-21

**Authors:** Christopher M. Wallis

**Affiliations:** Crop Diseases, Pests and Genetics Research Unit, USDA-ARS San Joaquin Valley Agricultural Sciences Center, 9611 S. Riverbend Ave, Parlier, CA 93648 USA

**Keywords:** Induced defense responses, Phenolics, Plant host resistance, Stilbenoids, Grapevine (*Vitis* spp.), Ring nematodes (*Mesocriconema xenoplax*)

## Abstract

**Objective:**

Ring nematodes can decrease vineyard productivity when plated in conditions favorable for their survival. Resistant rootstocks are available to combat harm due to ring nematodes, and compounds called phenolics were hypothesized as imparting this resistance. Therefore, this study measured phenolic compound levels in four different rootstocks and attempted to find associations with ring nematode populations. Furthermore, select phenolics called stilbenoids were tested in a bioassay to observe if these compounds affect ring nematode survival. This was part of a larger effort to assess the role of phenolics in protecting grapevines from nematodes and other pathogens or pests.

**Data description:**

This study was conducted over 2 years, 2018 and 2019, and phenolic levels were much greater in 2019 than 2018 likely due to uncontrolled differences in climatic controls. Ring nematode infected grapevines also did not have different phenolic compound levels than healthy controls. Bioassays of different stilbenoid polymers revealed no significant effects on ring nematode survival. These results suggest that analyzed root phenolic compounds were not involved in resistance or susceptibility to ring nematodes. These data should steer future researchers into analyzing other potential sources of nematode resistance.

## Objective

Ring nematodes, *Mesocriconema* (= *Criconema*) *xenoplax*, can have populations that reach very high levels and therefore can severely affect root functioning, with effects on overall plant health and yields [1–3]. Reductions in root growth and vine productivity can be observed by the third year after planting [4]. It is noted as the most difficult nematode to control and research [5].

Commercial vineyards are now commonly planted with *Vitis vinifera* cultivars grown as scions grafted onto rootstocks with wild *Vitis* spp., with rootstock cultivars targeted to combat specific pests such as nematodes [6–8]. Thus, the primary management option to limit ring nematode damage is resistant rootstocks [4, 9–11]. However, resistance mechanisms to combat ring nematodes are unclear, and overall knowledge examining ring nematode-grapevine host interactions is extremely limited [12–14]. In those studies, ring nematode feeding reduced grapevine nonstructural carbohydrate reserves and mineral nutrients [12–14].

However, effects of ring nematodes on grapevine production of defense-related compounds called phenolics also would be expected, especially a subclass called stilbenoids, as these may provide rootstock resistance against nematodes and other microbes [15]. Indeed, past studies have observed stilbenoids present in roots of grapevine and quantified high levels of five stilbenoid compounds: both stilbenoid monomers (resveratrol, piceatannol, and piceid) and dimers (ε-viniferin and δ-viniferin) [16]. Another study also has observed other polymers of stilbenoids including miyabenol C (a trimer), vitisin B and hopeaphenol (tetramers) [17–20].

Thus, this study attempted to measure phenolic compounds, including stilbenoids, and relate amounts to ring nematode counts and resistance. This was done to complement work examining effects of ring nematodes on carbohydrates and mineral nutrition, i.e. the experiments by Schreiner et al. [13, 14]. In addition, a bioassay was performed to assess whether different stilbenoid polymers could affect ring nematode survival. Results provided insights into the role of phenolics in plant-nematode interactions.

### Data description

Data provided cover two separate experimental replications, one in 2018 and one in 2019. Two grapevine rootstocks were analyzed per year (Schwartzman and St. George in 2018, O39-16 and self-rooted plants in 2019) with Cabernet Sauvignon as scions. All plants were established in 20 L pots with two parts sand to one part soil mix in a climate-controlled greenhouse kept at roughly 23 °C and receiving natural sunlight. Treatments were plants that did not receive nematodes and those that were inoculated with 1000 ring nematodes.

Data from these experiments involve nematode counts taken via the sugar flotation method from each pot 4 months after inoculations [14, 21] and chemical assessment of phenolic compounds taken from roots harvested at the same time. Phenolic compound qualification and quantification was made via a Shimadzu high-performance liquid chromatograph with a photodiode array detector, with compounds identified by further mass spectrometry and standard compound runs [22, 23]. Data are provided as Data file 1 (Table 1).

**Table 1:** Overview of data files/data sets

Label	Name of data file/data set	File types (file extension)	Data repository and identifier (DOI or accession number)
Data file 1	Ring nematode grapevine phenolic induction and sensitivity	MS excel file (.xlsx)	Ag Data Commons (https://doi.org/10.15482/USDA.ADC/1524667) [24]
Data file 2	Ring nematode phenolic project materials and methods	MS word file (.docx)	Ag Data Commons (https://doi.org/10.15482/USDA.ADC/1524667) [24]
Data file 3	Ring nematode phenolic induction and sensitivity data summaries	MS word file (.docx)	Ag Data Commons (https://doi.org/10.15482/USDA.ADC/1524667) [24]

In addition to these data, additional data are provided from a microplate bioassay that examined the potential of stilbenoids to affect ring nematode survival. Eight wells of one of two microplates has either 2.5, 5, 10, or 20 ppm of a stilbenoid monomer (piceid from Sigma-Aldrich, St. Louis, USA), dimer (ε-viniferin, Sigma), trimer (miyabenol C, from HPLC fraction collection), or tetramers (mixture of vitisin B and hopeaphenol, from fraction collection) in 200 µL of water with roughly ten ring nematodes in it. Water-only wells with ten nematodes were also present as controls. Data are provided in Data file 1 (Table 1).

More in-depth experimental methodology is provided as Data file 2 (Table 1). Lastly, a brief summary analyses of these data is provided as Data file 3 (Table 1). This includes a Figure that represents mean total phenolic, total flavonoid, and total stilbenoid levels (± SE) levels in roots collected from healthy and nematode-infected grapevines. Analyses of variance statistics and means separations by Tukey tests are provided for each year. Likewise, another summary Figure provides a summary of the mean ring nematode percent mortality (± SE) after 1 day in water amended with stilbenoid monomers (piceid), dimer (e-viniferin), trimer (miyabenol C), or tetramer (vitisin B/hopeaphenol).

### Limitations

This experiment was limited in the number of cultivars examined, as differences were apparent even among susceptible or resistant cultivars. Furthermore, there clearly was an effect of year, which is attributed to difference in greenhouse conditions as well as different incubation periods. Additional studies with greater control over these sources of variables could verify or result in different conclusions. Likewise, ring nematode inoculations should have been more carefully controlled, and possible involve re-inoculation of the soil every few weeks to ensure high, consistent levels of nematode pressure throughout the experiment. Bioassays also could have involved a greater variety of phenolic compounds and even specific stilbenoids, as those chosen might not necessary represent all other potential compounds that are present within grapevine roots. Unfortunately, many phenolics, and the majority of stilbenoid compounds, are unavailable commercially, and additional time-consuming isolations or syntheses are needed for these studies to proceed.

## Data Availability

The data described in this Data Note can be openly accessed at the USDA Ag Data Commons at: https://doi.org/10.15482/USDA.ADC/1524667.

## Abbreviations

HPLC: High-performance liquid chromatography
SE: Standard error
